# Why orthoptera fauna resist of pesticide? First experimental data of resistance phenomena

**DOI:** 10.1016/j.dib.2020.105659

**Published:** 2020-05-06

**Authors:** Djamel Brahimi, Lotfi Mesli, Abdelkader Rahmouni, Fatima Zohra Zeggai, Bachari Khaldoun, Redouane Chebout, Mohammed Belbachir

**Affiliations:** a University of Salhi Ahmed - Department of Sciences of Nature and Life Naama-45000, Algeria; b University of Abou Bakr Belkaid - Faculty of Sciences of Nature and Life and Sciences of the Earth and the Universe- Department of Ecology and Environment- Tlemcen-13000, Algeria; c Department of Chemistry, Laboratory of Polymer Chemistry, University of Oran1 Ahmed Benbella. BPN°1524 El'Menouer, 31000- Oran, Algeria; d Centre de Recherche Scientifique et Technique en Analyses Physico-chimiques (CRAPC), BP 38Bou-Ismail-RP 42004, Tipaza, Algeria

**Keywords:** Orthoptera, Chitin, Chitosan, Insect, Resistance, Pesticide, Arid region, Cuticle

## Abstract

Orthoptera are capable of threat of agriculture, human health and resists to all pesticides used. This problem is become an objectif of many research's. Pesticide resistance is the adaptation of insects to this materials resulting in decreased susceptibility to that chemical. In other hand, insects develop a resistance through natural selection such chemically transformation, physiological phenomena and genetic. In our study, natural chitin was extracted from cuticle of orthoptera insect (southern of Algeria) using a chemical strategy consists on hydrochloric acid, sodium hydroxide and hydrogen peroxide. The average yield of extracted chitin (96.95% w) indicates that the cuticles of orthoptera are a rich source of chitin. Cuticle exhibit a heterogeneous morphology characterized by a compact structure with well-defined fibrous. For extracted chitin and after demineralization, we can appreciate important changes in the surface of material. We observed round shaped black spots indicated that they are composed almost exclusively by K_2_O and CaO (cuticle) in the other hand we observed several white taches behind black spots, here we suggest that white taches present chitin extracted. The most resistant orthoptera are the ones to survive and transform their properties by chemical process such as transformation of chitin to chitosan and physiological development such as age. In this study, we have found that the first generation has a great resistance to insecticides. After insecticide application we observed a descendant's resistance decreased a larger because sensitive insects have been selectively killed. After repeated applications resistant insects may comprise the minority. Finally we can said, insecticide resistance can be found in many types and we can conclude that physiological resistance and chemical resistance coexist together and cannot separate. In the physiological resistance, the insect populations may develop the ability to avoid or reduce lethal insecticide exposure. In contrast, chemical resistance refers to modification mechanisms, including reduced cuticle penetration and decreased or increased target site sensitivity. The extracted chitin sample and chitosan were characterized by several characterizations such as X-ray diffraction, scanning electron microscopy SEM, FTIR and ^1^HRMN spectroscopy.

Specifications table

**Table:** 

Subject	Polymer chemistry, Chemical engineering
Specific subject area	Biological sciences, polymer chemistry, Chemical engineering, materials science, ecology.
Type of data	Table of sampling, Image, Figure of extracted and synthesized materials, extract yield.
How data were acquired	Morphology of product: SEM microgram Crystallographic properties :XRD Chemical composition: XRF. Structure of extracted and synthesized materials : ^1^HNMR
Data format	images, Tables and figures
Parameters for data collection	Materials prepared and synthesized was analyzed by their elemental composition as well as the morphological, crystalographic properties and structure. Parameters for the initial structures are provided in this article.
Description of data collection	Extracted chitin and synthesized chitosan from cuticle of orthoptera were used as new environmentally materials who can provide us with explanations on the resistance of orthoptera to pesticides.
Data source location	Republic algerian democratic and popular
**Data accessibility**	Data are supplied with this article
**Related research article**	BRAHIMI Djamel^1^*, MESLI Lotfi^1^ and RAHMOUNI Abdelkader^2^. Orthoptera fauna diversity in the arid region of Naama (Southern west of Algeria). Revue Agrobiologic.(2019)9(1) :1292-1301.

## Value of the data

• The data in this article will be informative to extracted of chitin and preparation of chitosan based on cuticle of orthoptera as raw material for study resistance phenomena of orthoptera at pesticides.
• Described dataset in this paper provides new idea to understand chemical and physiological phenomena explaining in when and how insect resist of pesticide. By using these data researchers can make comparisons with other resistance phenomena.
• Extraction of chitin and preparation of chitosan by these method employed in this Data article can be used as a reference for future studies to know the resistance of insects to pesticides.
• The Data obtained in this work can be effectively applied for all insects mostly of orthoptera.
• The data can be highlighted for further studies in development of better strategy for insect resistance to pesticide.

## 1 Data Description

**Scheme 1:**
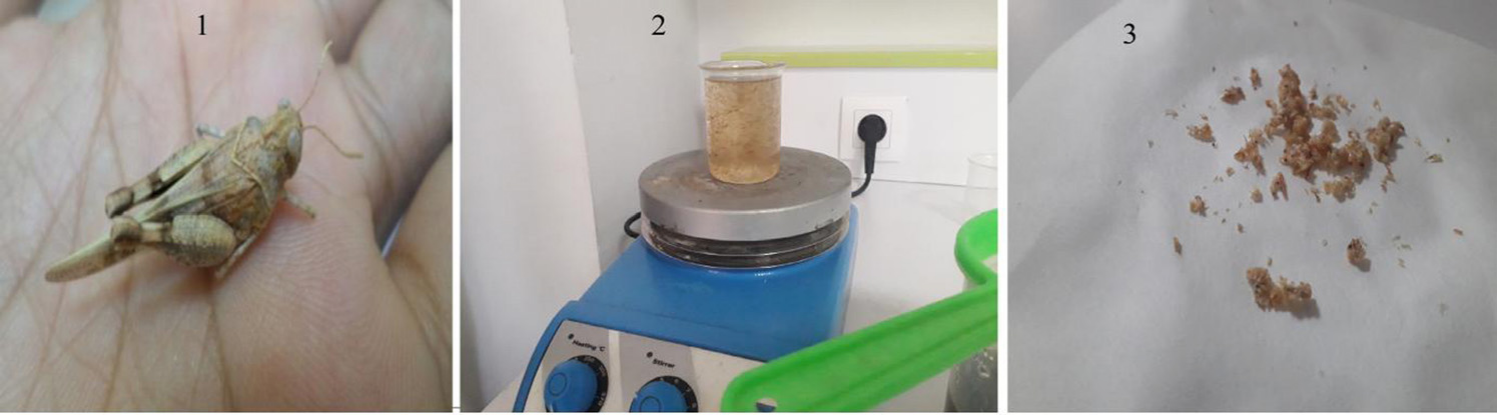
Describes of extracted chitin from cuticle of orthoptera in three steps

**Scheme 2:**
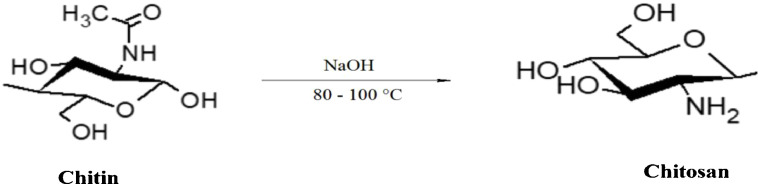
Describes of synthesized chitosan from cuticle of orthoptera fauna as raw material.

**Fig. 1:**
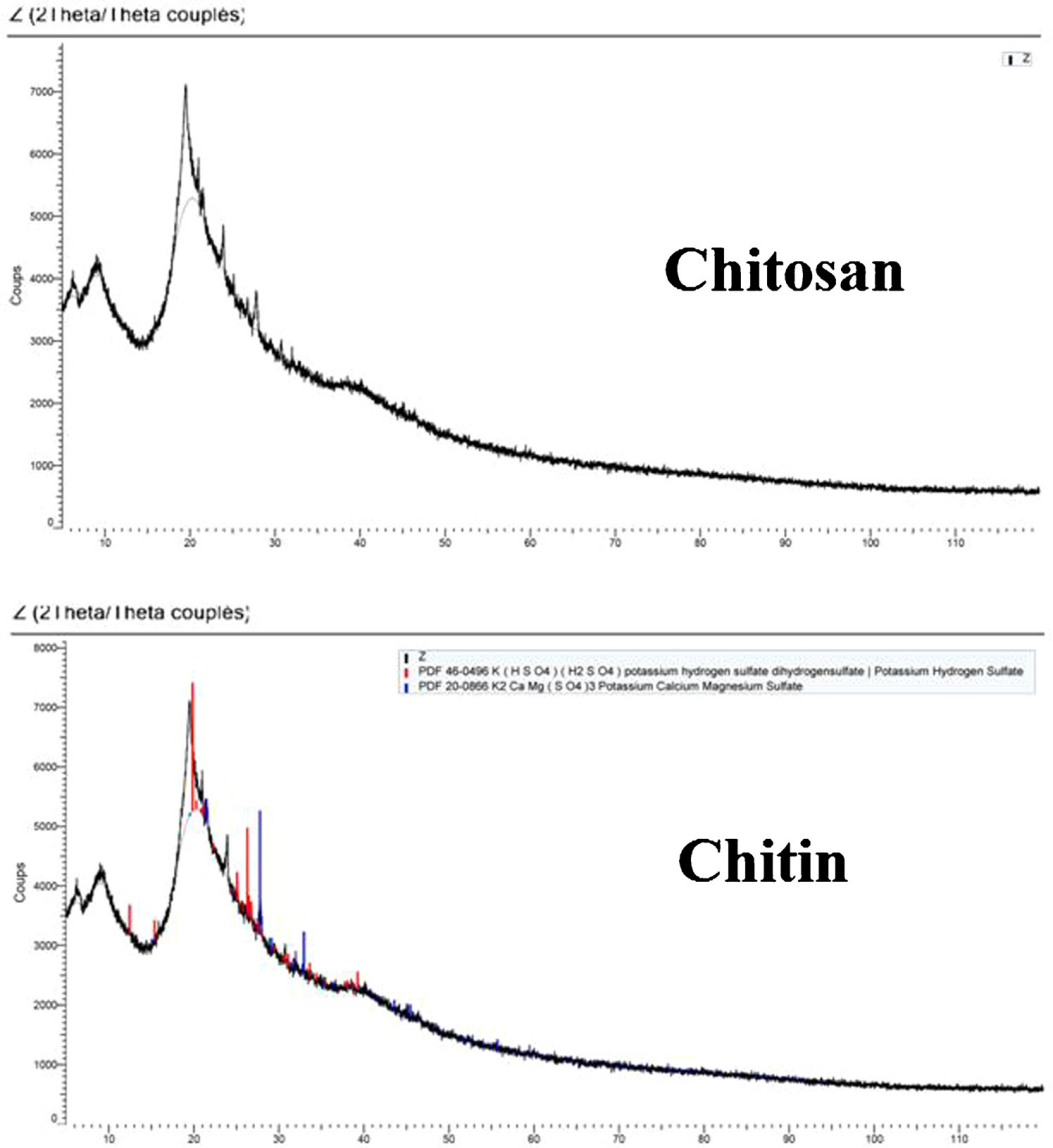
XRD pattern of extracted chitin and synthesized chitosan from cuticle of orthoptera.

**Fig. 2:**
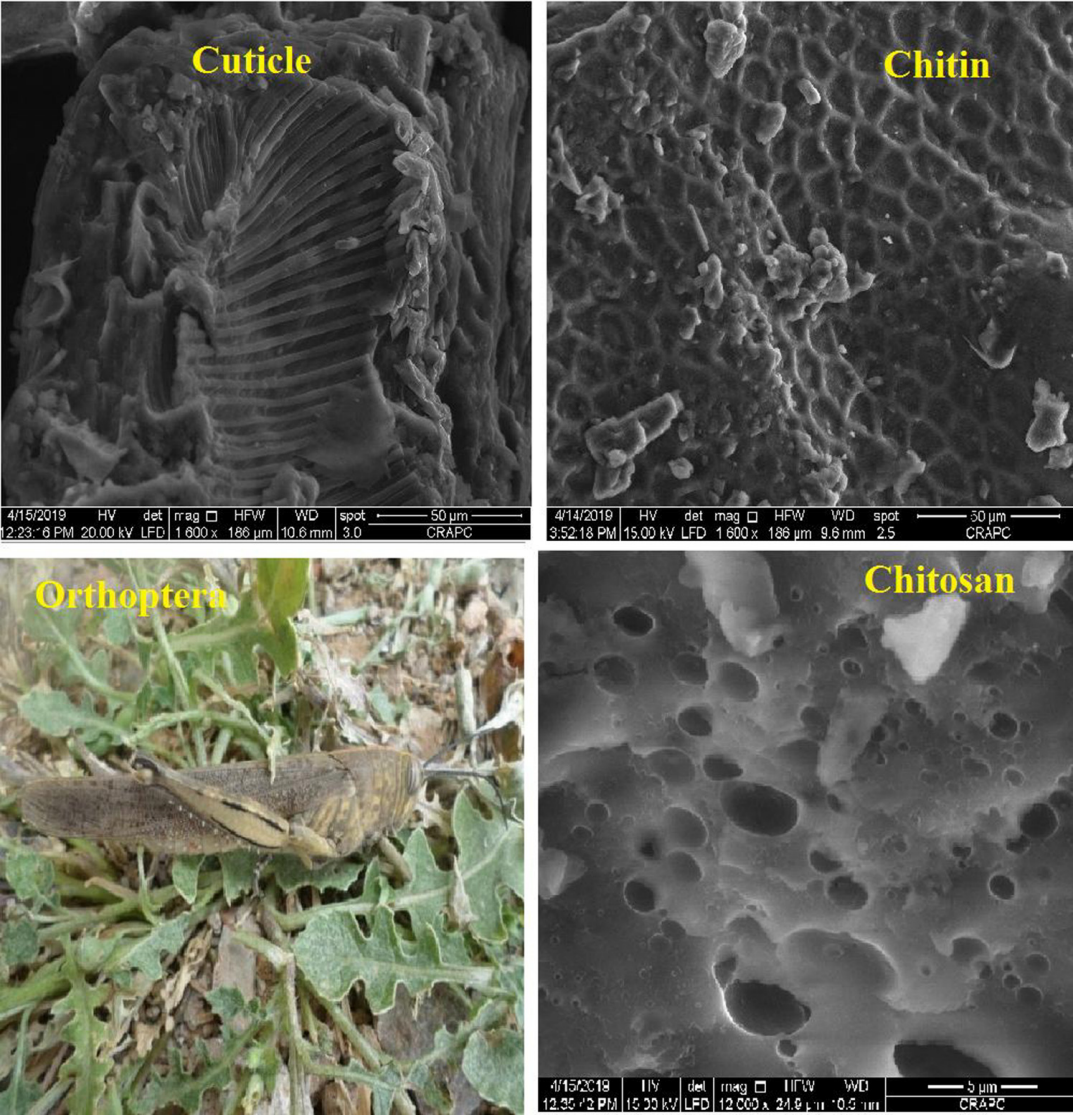
SEM micrographs of the cuticle, chitin and chitosan of orthoptera (southern of Algeria).

**Fig. 3:**
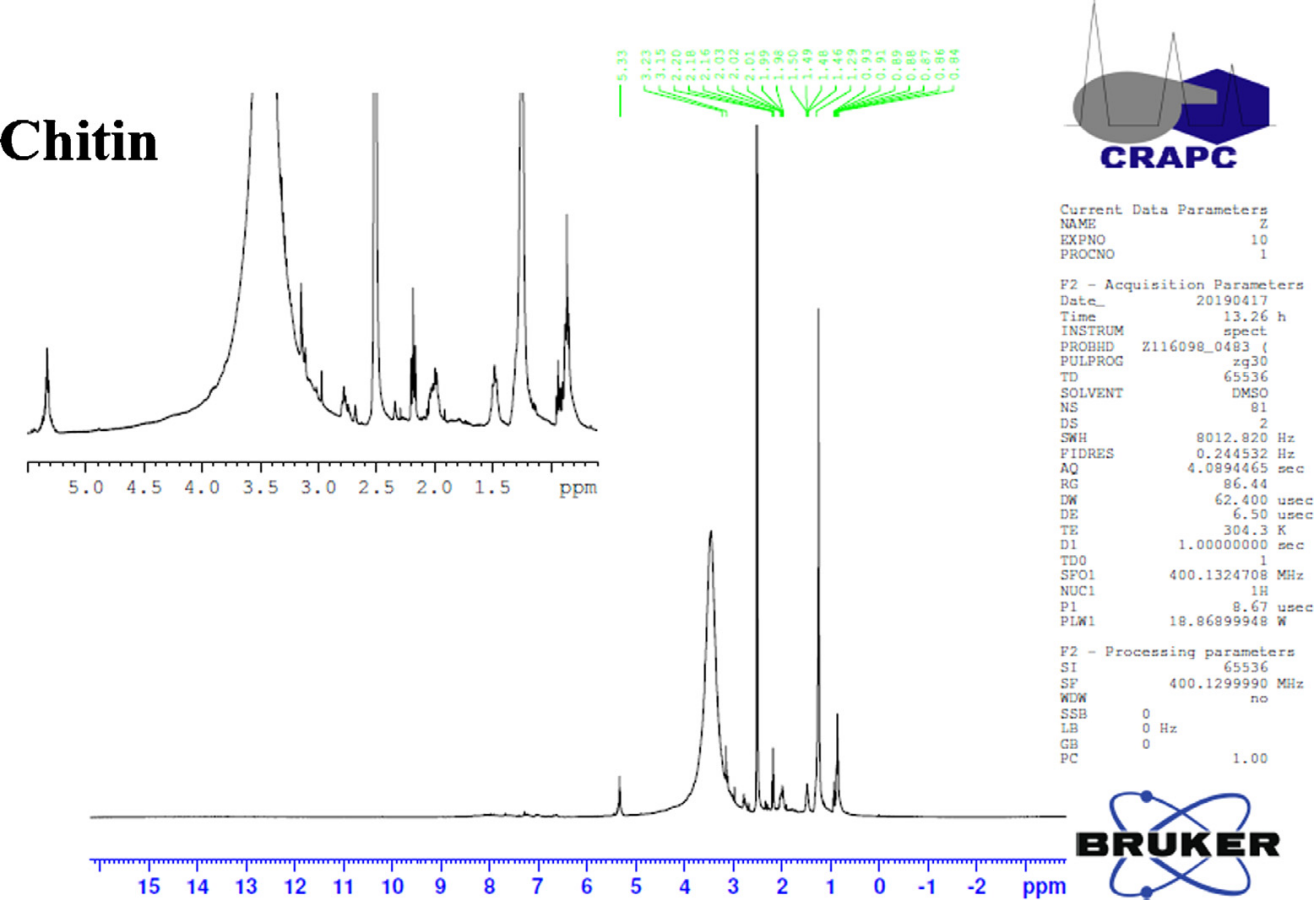
^1^H-NMR spectra of extracted chitin from cuticle of orthoptera in (DMSO).

**Fig. 4:**
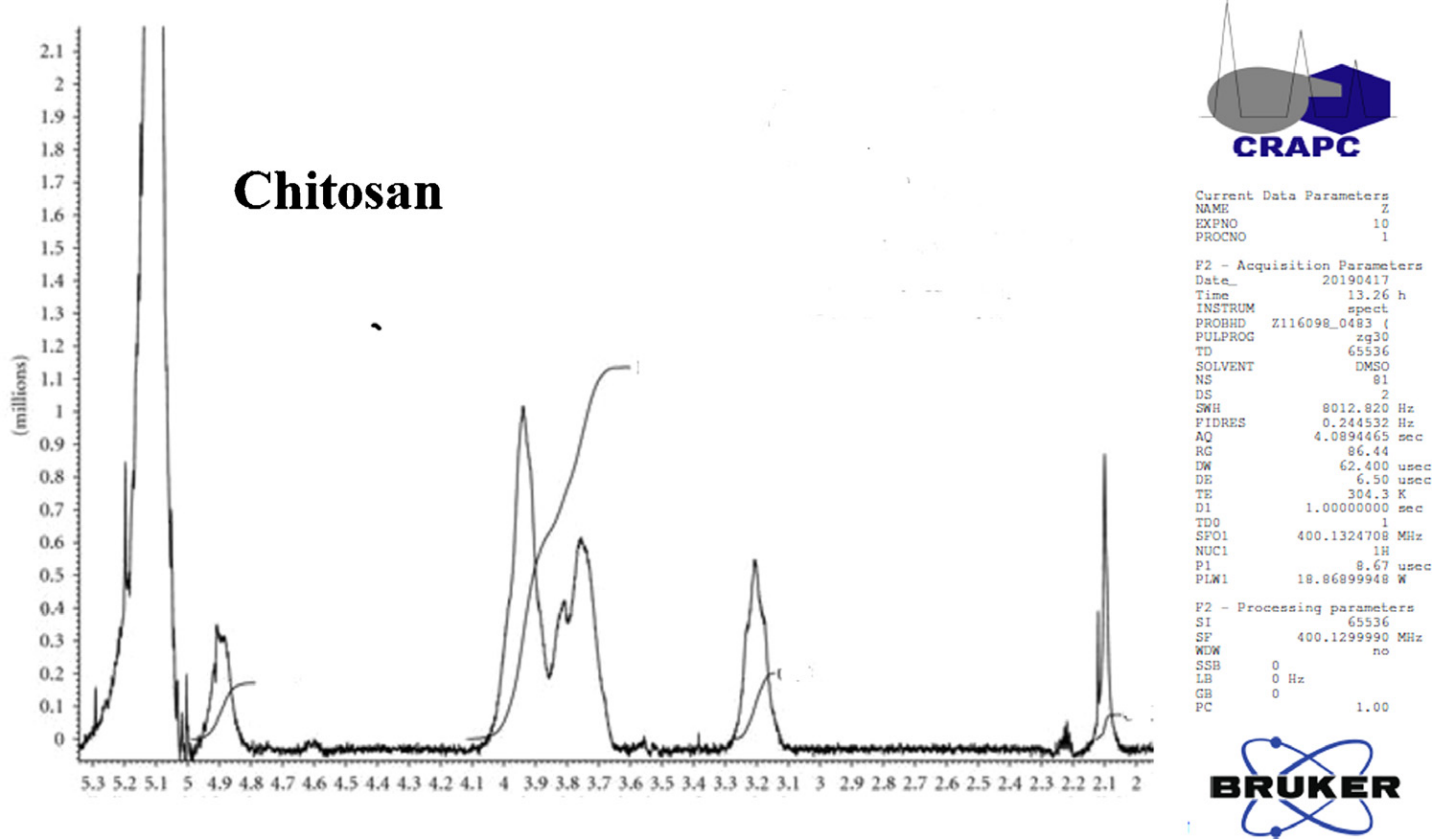
^1^H-NMR spectra of synthesized chitosan from natural chitin of orthoptera in (DMSO).

The Orthoptera for each station is studied with transects method. 13 samples were taken from August 2015 until August 2017. The number of mature individuals belonging to each locust species is counted separately [1]. Described dataset in this paper provides new idea to understand chemical and physiological phenomena how insect resist of pesticide [2]. The extracted chitin and synthesized chitosan of in these studies were confirmed by ^1^HNMR, XRF, XRD, and SEM [3]. Scheme. 1 describes of extracted chitin from cuticle of orthoptera. Scheme. 2 describes of synthesized chitosan from cuticle of orthoptera as raw materials. Table. 1 describes chemical composition of the chitin extracted from cuticle of orthoptera.). Tables. 2,3 and 4 describes duration of struggle and resistance percentage (%) resistance of orthoptera against the different insecticides. Fig. 1 describes the XRD pattern of extracted chitin and synthesized chitosan from cuticle of orthoptera. Fig. 2 describes SEM micrographs of the cuticle, chitin and chitosan of orthoptera (southern of Algeria). Fig. 3 describes ^1^HNMR spectra of extracted chitin and synthesized chitosan from cuticle of orthoptera. Fig. 4 describes ^1^H-NMR spectrum of prepared chitosan from natural chitin of orthoptera in (DMSO).

**Table 1:** Chemical composition of the chitin extracted from cuticle of orthoptera

No.	element	result (% by weight)	oxide	result (% by weight)
1	Al	0.482	Al2O3	0.9112
2	Si	3.03	Sio2	6.4716
3	P	7.87	P2O5	18.0222
4	S	6.13	SO3	15.3044
5	Cl	6.71	/	/
6	K	25.5	K2O	30.7407
7	Ca	8.57	CaO	11.9886
8	Fe	5.38	Fe2O3	7.6969
9	Ni	0.76	NiO	0.9672
10	Cu	0.31	CuO	0.3877
11	Zn	0.474	ZnO	0.5905
12	Br	0.213	/	/

**Table 2:** Duration of struggle and resistance percentage (%) of orthoptera against the organophosphates insecticide

Samples	Age	Insecticide	Duration of struggle (2015)	Resistance percentage (%)
1		Organophosphates	36 hours	35
2		Organophosphates	15 hours	21
3		Organophosphates	05 hours	2
4		Organophosphates	1 hour	0

**Table 3:** Duration of struggle and resistance percentage (%) of orthoptera against the fenitrothion insecticide.

Samples	Age	Insecticide	Duration of struggle(2017)	Resistance percentage (%)
1		Fenitrothion	48 hours	39
2		Fenitrothion	18 hours	20
3		Fenitrothion	8hours	02
4		Fenitrothion	2 hours	00

**Table 4:** Duration of struggle and resistance percentage (%) of orthoptera against the ethyl-chlorpyriphos insecticide.

Samples	Age	Insecticide	Duration of struggle(2019)	Resistance percentage (%)
1		Ethyl-chlorpyriphos	45 hours	46
2		Ethyl-chlorpyriphos	27 hours	19
3		Ethyl-chlorpyriphos	6 hours	04
4		Ethyl-chlorpyriphos	2 hours	00

## 2 Experimental Design, Materials, and Methods

### 2.1 Chemical and material

All reagents in this work were of analytical grade and used as received without further purification. Sodium hydroxide (NaOH) and chlorhydric acid (HCl) were used as initiator from sigma Aldrich (French). The cuticle of orthoptera used in this work came from a quarry located in Naama (southern west of Algeria) [4].

### 2.2 Study site

#### 2.2.1 Station of Mecheria

This station is located on the southern slopes of Jebel antar in north of the town of mecheria at (longitude 0° west and latitude 33° North). The vegetation covers in this station are (Stipa tenacissima, Peganum harmala and Aleppo pine) [5].

#### 2.2.2 Station of ben ammar

It is located at forty kilometer (40 Km) north of the mecheria city at ( longitude 0° west and latitude 33° north), the vegetation species in this station are (Stipa tenacissima, tamarix gallica and ziziphus lotus) [6].

#### 2.2.3 Wetland of Ain ben khelil

The resort is a wetland listed by ramsar. Its is localized at (longitude 0° west and latitude 33° north). The water of wetland concerned two hundred hectares surrounded by several units or peripheral areas; immediate area of water is characterized by tamarix and alfa formation. The gausses diagram and Ombrothermic bagnouls shows the dry period in the naama region is longer from april until October during the period (1985-2012). The rainfall climagramme emberger quotient (Q2) show that three stations located in the fresh winter upper arid area [7].

#### 2.2.4 Study of Orthoptera

The Study of orthoptera for each station is studied with transects methods. Thirteen samples were taken from August (2015) until August (2017). The number of mature individuals belonging to each locust species is counted separately. The collected specimens were preserved by both dry and wet preservation methods. The determination of orthoptera species is based on the chopard key (1943), and the acridoidea catalog of north west africa of Louveaux, A. & al. (1987) [8].

### 2.3 Extraction of chitin from cuticle of orthoptera

Orthoptera cuticle was suspended in 10% of chlorhydric acid (HCl) solution at room temperature for two hours. Deproteinization of cuticle was done by treating the demineralized cuticle with 10 % of sodium hydroxide (NaOH) at 100°C for two hours. After the incubation time, the residue product was washed to neutrality in running tap water and vacuum dried. Finally the product obtained was chitin (white powder, yield 96.95%) [9].

### 2.4 Chemical transformation of chitin to chitosan

Chitin obtained was deacetylatied by 50 % of sodium hydroxide( NaOH) solution at 80 to 100°C temperature for two hours, the obtained product was washed several time under mechanical stirring with water to neutrality, rinsed with deionized water then filtered, vacuum dried and grinded to obtained chitosan(white powder yielded 98.23 %) [10].

## Appendix Supplementary materials

Image, application 1
